# Proton pump inhibitors increase the chemosensitivity of patients with advanced colorectal cancer

**DOI:** 10.18632/oncotarget.18522

**Published:** 2017-06-16

**Authors:** Xiaoyu Wang, Chun Liu, Jiaqi Wang, Yue Fan, Zhenghua Wang, Yuanyuan Wang

**Affiliations:** ^1^ Third Ward of Oncology Department, The First Affiliated Hospital of Jinzhou Medical University, Jinzhou, Liaoning, 121001, China; ^2^ Department of Biochemistry, Liaoning University of Traditional Chinese Medicine, Shenyang, Liaoning, 110001, China; ^3^ Traditional Chinese Medicine Department, The First Affiliated Hospital of Jinzhou Medical University, Jinzhou, Liaoning, 121001, China; ^4^ Jinzhou Medical University, Jinzhou, Liaoning, 121001, China

**Keywords:** PPI, chemosensitization, 5-FU, FOLFOX, capeOx

## Abstract

Changes in pH can alter the uptake of chemotherapy drugs. Proton pump inhibitors (PPIs) may therefore increase the chemosensitivity of cancer cells and cytotoxicity of chemotherapeutic drugs by increasing their uptake. We investigated the chemosensitizing potential of PPIs in colorectal cancer (CRC). Our *in vitro* data show that the PPI pantoprazole increases the chemosensitivity of CRC HT29 and RKO cells to fluorouracil (5-FU). Our *in vivo* data demonstrate that pantoprazole also increases the ability of 5-FU to inhibit CRC tumor growth in mice. Importantly, a retrospective clinical study of CRC patients receiving the FOLFOX or CapeOx regimen indicates that PPIs increase the chemosensitivity of CRC patients. Patients who received the FOLFOX regimen with a PPI had better overall survival (OS) and progression-free survival (PFS) than patients who did not receive a PPI during FOLFOX chemotherapy. The incidence of nausea and vomiting was also lower in patients receiving a PPI with FOLFOX or CapeOx than in those who did not receive PPI. These results indicate that PPIs may be successfully incorporated into the FOLFOX regimen to increase the chemotherapeutic effect for CRC patients.

## INTRODUCTION

Colorectal cancer (CRC) is one of the most common malignant tumors [1–3]. Fluorouracil (5-FU) is the first-line anticancer drug in the treatment of CRC, but most patients with advanced or recurrent cases have poor prognosis because of developing chemoresistance [4]. The mechanisms of 5-FU chemoresistance in CRC remain unclear. Understanding of the mechanisms responsible for the 5-FU chemoresistance is important for developing novel therapeutic strategies for CRC.

Development of multiple drug resistance (MDR) has been associated with low pH in cancer cells [5]. High levels of H+ induce protonation and neutralization of chemotherapy drugs, thus decreasing their uptake by cancer cells [6]. The Warburg effect increases production of lactic acid, resulting in acidic microenvironment in cancer cells [7]. The acidic microenvironment then leads to the overexpressed proton pumps to avert acidification in cancer cells. Chemoresistance can be reversed by proton pump inhibitors (PPIs), which inhibit activity of H^+^−ATPases and drug retention outside of the cancer cells [8]. Recent studies have demonstrated that the sensitivity of B-cell tumors and breast cancer cells to chemotherapy drugs can be increased by PPIs [9, 10]. Thus, PPIs might represent an innovative class of chemosensitizers that increase the sensitivity of CRC cells to chemotherapy.

In this study, we tested the hypothesis that co-administration of a proton pump inhibitor may alter chemotherapy effectiveness. We used *in vitro* and *in vivo* approaches, as well as clinical data, to analyze the efficiency of PPIs in colorectal cancer.

## RESULTS

### Pantoprazole increases sensitivity of CRC cells to 5-FU *in vitro*

We evaluated efficiency of the PPI pantoprazole in increasing chemosensitivity of CRC cells to 5-FU using cell proliferation assay. First, we measured pH of the cell culture medium. After 24 hours in unbuffered medium, the pH of the extracellular microenvironment of CRC cells RKO and HT29 was 6.75 ± 0.05 and 6.92 ± 0.02, respectively. These conditions were suitable for the protonation effect of pantoprazole.

**Figure 1:**
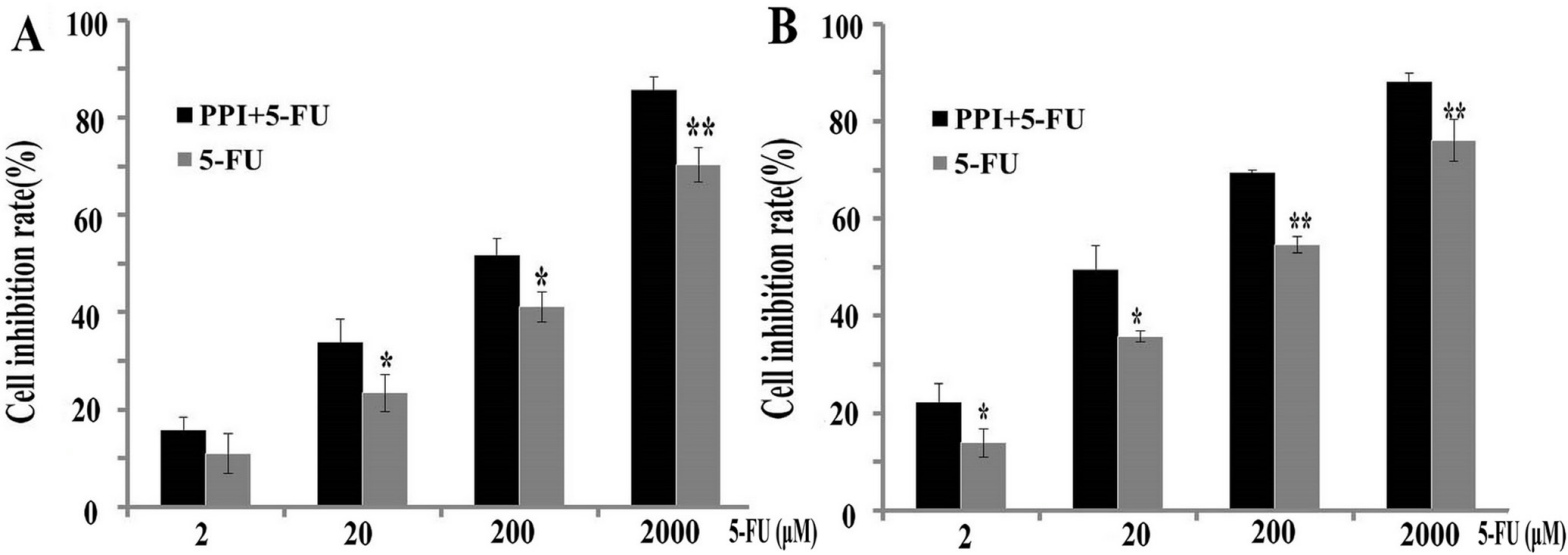
Pantoprazole increases sensitivity of CRC cells to 5-FU *in vitro* (**A**) Chemosensitivity to 5-FU (2, 20, 200, and 2000 μM) was compared with or without pantoprazole (50 μM) in RKO cells. The efficiency of 5-FU was higher in PPI group (*p < 0.05,**p < 0.01). (**B**) The chemosensitivity to 5-FU was compared with or without pantoprazole (50 μM) in HT29 cells. The efficiency of 5-FU was higher in PPI group (*p < 0.05,**p < 0.01) .

The chemosensitivity of CRC cells to 5-FU was remarkably increased by PPI (Figure 1). In HT29 cells, the cell inhibition rate of 5-FU at the lowest dose plus PPI was double in compared to non-PPI group (*p* = 0.04). In RKO cells, the cell inhibition rate of 5-FU at the higher dose was also increased in the PPI group compared to the non-PPI group (*p* = 0.04), suggesting that PPI may increase sensitivity of CRC tumors to 5-FU *in vivo*.

### Pantoprazole increases sensitivity of CRC tumors to 5-FU in mice

**Figure 2:**
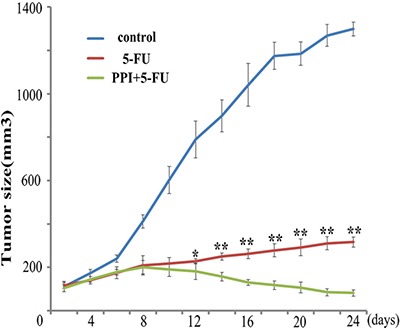
Pantoprazole increases sensitivity of CRC tumors to 5-FU in mice HT29 cells were injected s.c. into mice, and when the tumors were larger than 0.10 cm, each mouse in PPI group was injected i.p. with pantoprazole (30 mg/kg), and all mice were injected i.p. with 5-FU (5 mg/kg). The treatment of 5-FU and/or pantoprazole was repeated weekly for 4 weeks. Tumor size (mm3) was calculated as width^2^ × length/2 every two days. The tumor size between groups was analyzed by the Student's *t*-test. 5-FU alone inhibited the CRC tumor growth in mice, combination of 5-FU with pantoprazole had greater ability of inhibiting the tumor growth on the 12th day (*p* = 0.03). The tumor size of 5-FU plus PPI group was obviously smaller than the one of 5-FU group since the 12th day. (*p < 0.05, **p < 0.01).

The *in vivo* effect of pantoprazole on increasing sensitivity of CRC tumors to 5-FU was evaluated in mice injected with HT29 cells. As shown in Figure 2, while 5-FU alone inhibited the CRC tumor growth in mice, combination of 5-FU with pantoprazole had greater ability of inhibiting the tumor growth on the 12th day (*p* = 0.03). The tumor size of PPI and 5-FU group was obviously smaller than the one of 5-FU group since the 12th day (*p* < 0.05), suggesting that PPIs might increase chemosensitivity in CRC patients.

### PPIs increase chemosensitivity in CRC patients

Our retrospective chart review included 671 CRC patients; their characteristics are shown in Tables 1 and 2. In FOLFOX group, 259 patients received PPI and 48 patients did not receive PPI during chemotherapy. In CapeOx group, 215 patients received PPI and 149 patients did not receive PPI during chemotherapy. There was no statistical difference in age, gender distribution or cancer location (rectal or colon) between the two groups in the FOLFOX group. Except for nausea and vomiting, there was no difference in chemotherapy toxicity, such as myelosuppression, hepatotoxicity, hand foot syndrome, and diarrhea. The occurrence of nausea and vomiting in the PPI group (9%) was remarkably lower than in the non-PPI group (45%; *p* = 0.01). There was also no statistical difference in age, gender distribution and cancer location (rectal or colon) between the two groups in the CapeOx group. The only difference between the groups was nausea and vomiting, which was 15% in the PPI group, and 23% in the non-PPI group (*p* = 0.02).

**Table 1:** Baseline characteristics of colorectal cancer patients on FOLFOX taking PPI vs non-PPI

FOLFOX group	PPI	Non-PPI	*P* value
**Age**	51.9	51.3	0.07
**Gender**			
Male	175 (68%)	32 (67%)	0.07
Female	84 (32%)	16 (33%)	
**Location of cancer**			
Colon	151 (58%)	26 (54%)	0.11
Rectal	108 (42%)	22 (46%)	
**Toxicities**			
Myelosuppression	35 (14%)	14 (23%)	0.08
Hepatotoxicity	31 (12%)	15 (19%)	0.39
Hand foot syndrome	36 (14%)	15 (17%)	0.25
Diarrhea	31 (12%)	16 (29%)	0.11
Nausea and vomiting	24 (9%)	25 (45%)	0.01

**Table 2:** Baseline characteristics of colorectal cancer patients on CapeOx taking PPI vs non-PPI

FOLFOX group	PPI	Non-PPI	*P* value
**Age**	51.9	51.2	0.19
**Gender**			
Male	153 (73%)	105 (70%)	0.99
Female	62 (34%)	44 (30%)	
**Location of cancer**			
Colon	138 (64%)	95 (64%)	0.59
Rectal	77 (36%)	54 (36%)	
**Toxicities**			
Myelosuppression	36 (17%)	22 (15%)	0.67
Hepatotoxicity	42 (20%)	26 (17%)	0.48
Hand foot syndrome	30 (14%)	20 (13%)	0.61
Diarrhea	27 (13%)	24 (16%)	0.34
Nausea and vomiting	32 (15%)	34 (23%)	0.02

**Figure 3:**
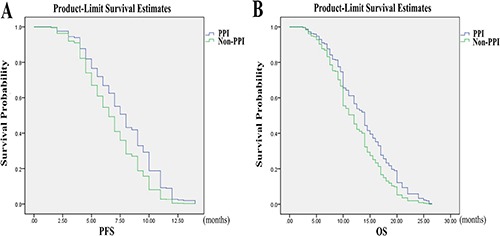
Progression-free survival (PFS) and overall survival (OS) by PPI use in colorectal cancer patients on FOLFOX therapy (**A**) In FOLFOX group, the PFS of patients taking PPI was statistically different compared with patients who did not receive PPI (*p* = 0.01, RR = 0.67, 95% CI = 1.10–2.05). (**B**) In FOLFOX group, the OS of patients taking PPI was statistically different compared with patients who did not receive PPI (*p* = 0.04, RR = 0.72, 95% CI = 1.02–1.90).

**Figure 4:**
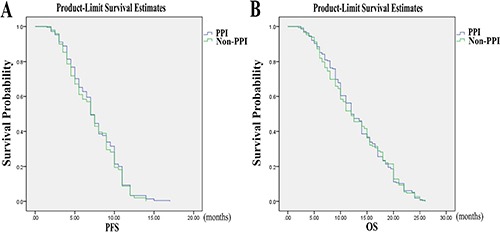
Progression-free survival (PFS) and overall survival (OS) by PPI use in colorectal cancer patients on CapeOx therapy There was no statistical difference in PFS and OS between the patients with or without PPI in CapeOx group (PFS: *p* = 0.52,OS: *p* = 0.98).

Progression-free survival (PFS) and overall survival (OS) in the FOLFOX group differed between patients taking PPI and patients who did not receive PPI (OS: *p* = 0.04; RR=0.72, 95% CI=1.02–1.90; PFS: *p* = 0.01; RR=0.67, 95% CI=1.10–2.05; Table 3). OS and PFS of the patients receiving 5-FU plus PPI did better than those with 5-FU alone according to RR. The statistical difference was also revealed using the Kaplan-Meier curves (Figure 3). However, age, gender, and performance status (PS) did not differ between patients taking PPI and patients who did not receive PPI, indicating that age, gender, and PS do not affect survival. There was no statistical difference in age, gender, and PS between patients with or without PPI in the CapeOx group. The use of PPI did not affect survival of patients in the CapeOx group (PFS: *p* = 0.52; OS: *p* = 0.98; Table 3). The Kaplan-Meier curves confirmed the results (Figure 4).

**Table 3:** The multivariate analysis of prognostic factors weighed by Cox's proportional hazard model

FOLFOX group		RR	95%CI	*P* Value	XELOX group		*P* Value
**PFS**	**Age**			0.32	**PFS**	**Age**	0.11
	**Gender**			0.64		**Gender**	0.71
	**PS**			0.56		**PS**	0.31
	**PPI**	0.67	1.10–2.05	0.01		**PPI**	0.52
**OS**	**Age**			0.17	**OS**	**Age**	0.31
	**Gender**			0.66		**Gender**	0.73
	**PS**			0.44		**PS**	0.81
	**PPI**	0.72	1.02–1.90	0.04		**PPI**	0.98

RR = relative risk, CI = confidence interval, PFS = progression-free survival, PS = performance status, PPI = proton pump inhibitor, OS = overall survival.

## DISCUSSION

Acidic microenvironment may induce tumor growth [11]. *In vitro* and *in vivo* studies have shown that acidic microenvironment increases chemoresistance of cancer cells, suggesting that increasing the pH of tumor cells by using PPIs can make cancer cells more sensitive to chemotherapy. Indeed, recent studies have indicated that PPIs may increase sensitivity to chemotherapy in cancer patients [12–16].

Our *in vitro* data have demonstrated that PPI increases chemosensitivity of CRC cells to 5-FU, and that the PPI pantoprazole may be activated due to the acidic microenvironment of CRC cells. Although HT29 and RKO colorectal cancer cells could be effectively killed by 5-FU alone, the efficiency of 5-FU was increased by pantoprazole. Our *in vivo* data have shown that pantoprazole also increases efficiency of 5-FU in inhibiting CRC tumor growth in mice. Importantly, our retrospective clinical study indicates that PPIs also increase the 5-FU efficiency in CRC patients. The patients who used FOLFOX regimen along with PPI had better OS and PFS compared with the patients who did not receive PPI during FOLFOX chemotherapy. However, there was no statistical difference in OS and PFS in patients receiving the CapeOx regimen. One possible explanation may be the oral anti-cancer drug capecitabine used in the CapeOx regimen. Some drugs, such as capecitabine, need acidic pH in order to be dissolved and absorbed [17, 18]. Thus, acid reducers, such as PPIs may decrease the absorption of oral drugs such as capecitabine, resulting in their decreased efficiency. Different chemotherapy drugs and drug combinations have different toxicities [19, 20]. Our study showed that the occurrence of nausea and vomiting in the PPI group was remarkably lower than in the non-PPI group, indicating that PPIs help patients decrease the occurrence of nausea and vomiting.

This is the first study demonstrating that PPIs increase chemosensitivity of CRC cells *in vitro*, *in vivo*, and in a clinical setting. Together, our results indicate that PPIs may be successfully incorporated into the FOLFOX regimen to increase the chemotherapeutic effect for colorectal cancer patients. Our study still has some limitations and the molecular mechanism of PPI increasing chemosensitivity needs to be further studied. Meanwhile the large prospective clinical controlled study is also needed.

## MATERIALS AND METHODS

### Cell culture

Human colorectal cancer RKO and HT29 cells, obtained from ATCC (Rockville, Maryland, USA), were grown in RPMI-1640 medium supplemented with 10% heat-inactivated fetal bovine serum (FBS, Gibco, USA), 100 μg/ml streptomycin and 100 U/ml penicillin at a humidity of 90% containing 5% CO_2_ at 37°C. Cells grew as a single cell layer attached to specially treated plastic surfaces, and were sub-cultured 2 to 3 times a week. Cells during exponential growth phase were used in the experiments.

### Cell proliferation assay

RKO and HT29 cells were plated at 1 × 10^4^ cells per well in 100 μl of RPMI-1640 with 10% FBS in 96-well plates for 24 h. The cells were then transferred to a new medium containing pantoprazole (50 μM; Nycomed, Berlin, Germany) and 5-FU (2, 20, 200, and 2000 μM) and cultured at a humidity of 90% at 5% CO_2_ and 37°C for 72 h. 10 μl of CCK (5 mg/l) was added to each well, and cells were incubated for additional 4 hours. The absorbance (A) was measured using a multi-well spectrophotometer (Bio-Tek, XL-80) at 450 nm. The proliferation rate was calculated as follows: Cell inhibition rate (%) = 1- A of the treated wells/ A of the control wells (5-FU 0 μM, PPI 0 μM)×100.

### Animal experiment

4–5 weeks old female BALB/c mice (Guangzhou, China) were housed under pathogen free conditions and fed *ad libitum*. About 1 × 10^6^ HT29 cells were injected subcutaneously (s.c.) into each mouse. When the tumors reached 0.10 cm (about 1 week after injection), mice in PPI group were injected intraperitoneally (i.p.) with pantoprazole (30 mg/kg) and 5-FU (5 mg/kg); the treatment with pantoprazole and 5-FU was repeated weekly for 4 weeks. Tumor size (mm3) was calculated as width^2^ × length/2 every two days. According to standard clinical criteria, which included general illness, rough hair coat, weight loss (> 20%) and oversized tumor (> 1 cm), the end-point was morbidity. There were at least four mice in each group. Physiological indexes, such as diarrhea, hair color, and weight, were detected throughout the experiment. The animals were treated according to the European Council Directive 86/609/EEC.

### Clinical research

In this retrospective study, we selected advanced stage (stage IV) CRC patients at the First Affiliated Hospital of Jinzhou Medical University, Jinzhou, China, from January 1, 2010 to December 31, 2014. Data was collected from electronic and paper medical records.

All patients were more than 18 years old and did not receive surgical treatment. The first regimen they received was FOLFOX (Oxaliplatin 85 mg/m^2^ IV, day 1; Leucovorin 400 mg/m^2^ IV, day 1; 5-FU 400 mg/m^2^ IV bolus on day 1; then 1200 mg/m^2^/day ×2 days continuous infusion over 46–48 hours; repeated every 2 weeks.) Alternatively, the patients received CapeOx regimen (Oxaliplatin 130 mg/m^2^ over 2 hours, day 1; Capecitabine 1000 mg/m^2^ twice daily, days 1–14; every 3 weeks). Patients who received concurrent radiotherapy were excluded. Clinical data included gender, birth, performance status (PS), toxicity, PPI use, PFS, and OS. The FOLFOX and CapeOx groups were each divided into non-PPI and PPI groups. The patients who received PPI during chemotherapy belonged to the PPI group. The method of entering variables into the multivariate model was forward method. The data of non-PPI and PPI groups were compared in FOLFOX group. Then, the data of non-PPI and PPI groups were compared in CapeOx group. The patients were followed up for 5 years.

### Statistical analysis

SPSS 19.0 software program was used to evaluate the statistics. All data are shown as the mean ± standard deviation (S.D.). The statistical analysis of cell and animal experiments was done by the Student's *t*-test. OS and PFS of the patients were analyzed by Kaplan-Meier curves and Cox's proportional hazard model to compare patients receiving chemotherapy and PPI, with patients receiving chemotherapy without PPI. Results were considered as statistically significant when *p* < 0.05. All assays in cell and animal experiments were carried out in triplicates.

### Statement of ethics

Ethics approval was obtained from the Health Research Ethics Board of the First Affiliated Hospital of Jinzhou Medical University. Given the nature of a retrospective chart review, informed consent was not required.

## Abbreviations

RR: relative risk
CI: confidence interval
PFS: progression-free survival
PS: performance status
PPI: proton pump inhibitor
OS: overall survival
